# Promoting hand hygiene program via social media

**DOI:** 10.1186/2047-2994-4-S1-P165

**Published:** 2015-06-16

**Authors:** S-C Pan, K-L Tien, W-H Sheng, K-T Chien, Y-C Chen, S-C Chang

**Affiliations:** 1 Department of Internal Medicine, National Taiwan University Hospital, Taipei, Taiwan, Province of China; 2 Center for Infection Control, National Taiwan University Hospital, Taiwan, Province of China; 3 Information Technology Office, National Taiwan University Hospital, Taiwan, Province of China

## Background

Global hand hygiene (HH) campaign has been promoted since 2004 to protect the patient safety. On World HH day (May 5), 2014, a video campaign was launched in National Taiwan University Hospital (NTUH), Taiwan. We aimed to evaluate the effect of different social media on promoting the HH program.

## Methods and materials

One 3 minutes-long video of HH campaign in 8 language was posted on the YouTube. The Chinese version had been promoted through three platforms, including the official hospital website, hospital group e-mail, and the Facebook site of one famous internet celebrity, who was also 6th grade medical student of the school the hospital affiliated to. The video traffic of the Chinese version during May 5, to Dec, 31, 2014 was analyzed via Google Analytics. The HH compliance was observed and compared in Nov. of 2013 and Nov. 2014, separately.

## Results

During the observed period, 5,252 times the video was viewed, mainly on the Chinese version (3,509/5,252, 66.8%). The cost of the video was 6,250 USD and the cost per clink was 1.2 USD. For the hospital level, the official website of NTUH had 24,000 subscribers and 151 linked to the video. The connection rate was 0.6% (151/24,000). As to the hospital-group e-mail, 9,967 peoples received the e-mail and 91 receiver opened the link (connection rate 0.9%, 91/9,967). There were no further feedback from the e-mail system or website. For the personal level, there were 13,080 impressions from the internet celebrity’s Facebook site and 807 had linked into the video. The connection rate as 6.2% (807/13,080) was significantly higher than the hospital website and group e-mail (both P value<0.001). 525 persons (4.0%, 525/13,080) had pressed “Like” for the HH campaign and 21 (0.2%, 21/13,080) persons further shared the video. The hospital-wide HH compliance was 83.7% (473/565) in 2013 and 86.7 % (589/679) in 2014 (P=0.13).

## Conclusions

We succeeded the HH compliance via a video campaign in 2014. For the Net Generation, social media as Facebook had provided significantly high connection rate. It revealed that the information transmission may not be depended on the hierarchy within the hospital. The information leader in novel social media, like Facebook, could be a strong support for future HH promoting program.

## Disclosure of interest

None declared.

